# Lung Cancer Surgery after Treatment with Anti-PD1/PD-L1 Immunotherapy for Non-Small-Cell Lung Cancer: A Case—Cohort Study

**DOI:** 10.3390/cancers13194915

**Published:** 2021-09-30

**Authors:** Kinan El Husseini, Nicolas Piton, Marielle De Marchi, Antoine Grégoire, Roman Vion, Pierre Blavier, Luc Thiberville, Jean-Marc Baste, Florian Guisier

**Affiliations:** 1 CHU Rouen, Department of Pneumology, F-76000 Rouen, France; kinan.el-husseini@univ-rouen.fr (K.E.H.); marielle.demarchi@orange.fr (M.D.M.); antoine.gregoire@chu-rouen.fr (A.G.); 2 Normandie Université, UNIROUEN, INSERM U1245, Normandy Centre for Genomic and Personalized Medicine and CHU Rouen, Department of Pathology, F-76000 Rouen, France; nicolas.piton@chu-rouen.fr; 3 CH Elbeuf, Department of Pneumology, F-76503 Saint-Aubin-lès-Elbeuf, France; 4 CH Dieppe, Department of Pneumology, F-76202 Dieppe, France; 5 Centre Henri Becquerel, Department of Medical Oncology, F-76000 Rouen, France; roman.vion@chb.unicancer.fr; 6 CHU Rouen, Departments of Biostatistics and Public Health, F-76000 Rouen, France; pierre.blavier@chu-rouen.fr; 7 Normandie Université, UNIROUEN, LITIS Lab QuantIF Team EA4108, CHU Rouen, Department of Pneumology and Inserm CIC-CRB 1404, F-76000 Rouen, France; luc.thiberville@univ-rouen.fr; 8 CHU Rouen, Department of Thoracic Surgery, F-76000 Rouen, France; jean-marc.baste@chu-rouen.fr

**Keywords:** non-small-cell lung cancer, immune checkpoint inhibitors, immunotherapy, anti-PD1/PD-L1, lung resection, safety

## Abstract

**Simple Summary:**

The scope of indications for immune checkpoint inhibitors (ICIs) in non-small-cell lung cancer is growing, and an increasing number of patients are undergoing lung resection surgery after ICI treatment, with some technical difficulties being reported. The aim of our study was to determine if preoperative ICIs were associated with more difficult lung surgeries or poorer perioperative outcomes compared to surgeries performed after induction chemotherapy. We confirmed that ICIs were associated with tissue fibrosis and inflammation, particularly in centrally located lung tumours, although this did not translate to higher rates of perioperative morbidity. There was no 90-day mortality. We also found higher rates of major pathological response to pre-operative treatment in the ICI cohort and higher disease-free survival. Our findings further support the safety of lung resection in patients following preoperative ICIs.

**Abstract:**

Background: Immune checkpoint inhibitors (ICIs) are the standard of care for non-resectable non-small-cell lung cancer and are under investigation for resectable disease. Some authors have reported difficulties during lung surgery following ICI treatment. This retrospective study investigated the perioperative outcomes of lung resection in patients with preoperative ICI. Methods: Patients with major lung resection after receiving ICIs were included as cases and were compared to patients who received preoperative chemotherapy without ICI. Surgical, clinical, and imaging data were collected. Results: A total of 25 patients were included in the ICI group, and 34 were included in the control group. The ICI patients received five (2–18) infusions of ICI (80% with pembrolizumab). Indications for surgery varied widely across groups (*p* < 0.01). Major pathological response was achieved in 44% of ICI patients and 23.5% of the control group (*p* = 0.049). Surgery reports showed a higher rate of tissue fibrosis/inflammation in the ICI group (*p* < 0.01), mostly in centrally located tumours (7/13, 53.8% vs. 3/11, 27.3% of distal tumours, *p* = 0.24), with no difference in operating time (*p* = 0.81) nor more conversions (*p* = 0.46) or perioperative complications (*p* = 0.94). There was no 90-day mortality. Disease-free survival was higher in the ICI group (HR = 0.30 (0.13–0.71), *p* = 0.02). Conclusions: This study further supports the safety and feasibility of lung resection in patients following preoperative treatment with ICI.

## 1. Introduction

In the last ten years, the development of immune checkpoint inhibitors (ICIs) has revolutionized the treatment landscape of non-small-cell lung cancers (NSCLC) in both the metastatic and locally advanced stages. These monoclonal antibodies target molecules involved in the co-inhibition pathways of T lymphocyte activation, such as the *programmed death receptor-1* (PD-1)/ligand (PD-L1) or the *cytotoxic T-lymphocyte associated protein 4* (CTLA-4). By blocking the inhibitory signals present on the surface of cancer cells, ICIs are able to reverse tumor-induced T-cell anergy and to restore a competent anti-neoplastic immune response [1].

Studies of ICIs in NSCLC and melanoma have suggested that a subset of patients with metastatic cancer could achieve long-term disease control [2]. Hindsight afforded by the continuous follow-up of metastatic or advanced NSCLC patients who participated in the first large scale ICI trials has confirmed the significant increase in overall survival (OS) compared to standard chemotherapy in a population with a historically dismal prognosis. For example, data from the phase Ib KEYNOTE-001 study investigating pembrolizumab (anti-PD-1) monotherapy in advanced NSCLC found estimated 5-year OS rates of 23.2% and 15.5% among treatment-naïve and previously treated patients, respectively [3]. Similar findings were reported in clinical trials of nivolumab (anti-PD-1) in previously treated advanced NSCLC, with a 5-year OS of 16% [4]. These results are a meaningful improvement over the 5-year OS rate of 5.5% that was achieved with standard-of-care chemotherapy prior to the development of ICIs in the same setting [5].

ICIs have also become pivotal in the management of locally advanced NSCLC following the PACIFIC trial, which found a progression-free-survival (PFS) of 16.8 months among patients with unresectable stage III NSCLC treated with chemoradiotherapy and adjuvant durvalumab (anti-PD-L1) compared to 5.6 months in the placebo arm [6]. Today, ICI treatment is the standard of care for virtually all unresectable NSCLC patients without molecular alterations eligible that are for targeted therapies [7]. ICIs are also being investigated for use as a monotherapy or combined with chemotherapy in the adjuvant/neoadjuvant settings for resectable disease [8,9], which accounts for 20–30% of NSCLC cases [10].

According to current guidelines, ICIs have yet to be recommended in resectable NSCLC [11]. However, in the real-world setting, an increasing fraction of patients treated with ICIs for stage III or IV NSCLC undergoes lung cancer surgery. Among stage IV NSCLC, lung cancer surgery after ICI treatment may be proposed in two main settings: (i) the multimodal management of oligometastatic disease and (ii) residual disease ablation after partial tumour response to ICI treatment. Even if it has not been prospectively validated, local ablative treatment, including surgery, may be proposed for selected oligometastatic NSCLC patients, either as a front-line multimodal strategy or in long-term responders to immunotherapy. Additionally, some patients with potentially resectable stage III disease are being treated with chemo-immunotherapy before lung cancer surgery is performed. Oligometastatic stage IV NSCLC is widely recognized as metastatic NSCLC with five or less metastatic sites involving no more than three organs [12]. When all tumour sites are amenable to local ablative treatment, patients may benefit from a combined approach consisting of the local radical treatment of tumoral sites along with systemic therapy [13].

In any case, the safety and technical aspects of lung resections following ICI treatment have rarely been reported [14,15,16]. Indeed, although ICIs are characterized by a better safety profile compared to standard chemotherapy with generally milder and more manageable adverse events [3,17], surgical interventions on these patients may be marred by technical difficulties related to peri-tumoral inflammation induced by immunotherapy [15,18]. This inflammation may increase tissue adhesion and therefore surgical difficulties. Moreover, the foreseeable advent of neoadjuvant immunotherapy in the near future could further increase the number of patients referred for lung resection following ICI treatment, highlighting the need for additional safety data. Hence, the aim of this case–control study was to describe the safety and feasibility of lung resection in a cohort of patients who previously received ICI treatment for NSCLC in any setting, while comparing them to patients who received surgery following a standard induction treatment with chemotherapy alone. Our hypothesis was that prior ICI treatment could increase tissue adhesion and therefore cause more surgical difficulties and complications.

## 2. Materials and Methods

### 2.1. Study Population

We used a combination of methods to identify all consecutive patients aged 18 and older who had received treatment with ICI for NSCLC prior to a major lung resection performed between 1 January 2015 and 1st February 2021 (“ICI group”). First, we cross-referenced all patients who received ICI during this interval at our institution (*Chimio*© software, Computer-Engineering, Paris, France) with the surgical database maintained by our Thoracic Surgery department as part of a nationwide data collection initiative (Epithor register) [19]. Common entries were screened to identify patients who had received ICI before surgery. In parallel, electronic health records were queried to find all patients with a mention of “ypTNM” in a pathologic report and an ICD-10 coding for lung resection. 

This allowed us to identify patients who had undergone lung cancer surgery at our institution and who had received pre-surgery treatment in other centers. From this secondary list, additional information was obtained from partner centers. Overall, patients who received pre-operative ICI treatment (with or without chemotherapy) were included in the ICI group, whereas patients who received chemotherapy-based induction regimens were included as controls. All patients underwent a standard preoperative staging workup, comprising pulmonary function tests (PFT), exercise testing when indicated, pretreatment tumour biopsy, chest contrast-enhanced computed tomography and/or PET/CT scan, and invasive nodal staging as indicated. Patients also underwent brain CT-scan or MRI when clinically indicated. Resection of the primary tumour and lymphadenectomy was performed according to institutional standards. 

In the screening process, patients were excluded because they did not meet the inclusion/exclusion criteria: (i) after the EPITHOR database and our institution’s chemotherapy database cross-referencing, 46 patients were excluded because they actually received ICIs after surgery and not before; (ii) following the “ypTNM” text query in our institution’s clinical database, 18 patients were excluded because they were duplicates, 20 patients were excluded because no information was available about their preoperative systemic treatment (these patients had lung surgery in our center after referral from other centers), 12 patients were excluded because the surgery that they received was not a lung resection (mainly pleural biopsies), and 7 other patients were excluded because their surgery was performed in another center, with no information available about any peri-operative complications.

### 2.2. Data Collection

Clinical data were extracted from the institutional electronic health record systems, including demographic variables, anthropometry (height, weight), co-morbid conditions, PFT, exercise testing results, and pathological reports. Surgical intervention data were retrieved from a detailed review of the surgical reports. Additionally, surgical outcomes and peri-operative complications were extracted from the Epithor register, in which complications are recorded and graded exhaustively according to the Clavien–Dindo classification [20,21] alongside data on intervention type, duration, hospital stay length, and follow-up. CT scans performed at cancer diagnostic, at the time of ICI treatment initiation (baseline), and immediately prior to surgery were reviewed. Clinical staging was determined using the 8th edition of the TNM Classification System for Thoracic Cancers published by the Union for International Cancer Control and the American Joint Committee on Cancer [22]. Topography of the target lesion, i.e., the lung tumour that was resected during surgery, was recorded: centrally located tumours close to the hilum/mediastinum were defined as “proximal” as opposed to “distal” tumours, which were located more peripherally and “sulcus” tumours, which were located in the superior pulmonary sulcus.

### 2.3. Statistical Analysis

Quantitative variables are presented as either median (inter-quartile range) or mean ± standard deviation, and qualitative variables are presented as absolute and relative frequencies. Comparisons of quantitative and qualitative variables were made using the Mann–Whitney test and the chi-squared or the Fisher exact test, respectively. Kaplan–Meier survival curves were calculated and compared using the log-rank test. All tests were two-sided, with *p* = 0.05 indicating statistical significance. We applied Bonferroni correction for multiple comparisons where applicable. All statistical analyses were performed using *GraphPad Prism* version 8.4.2 software for Windows (GraphPad Software, San Diego, CA, USA) and R Statistical Software (version 4.0.5; R Foundation for Statistical Computing, Vienna, Austria). 

## 3. Results

### 3.1. Study Population

**Figure 1:**
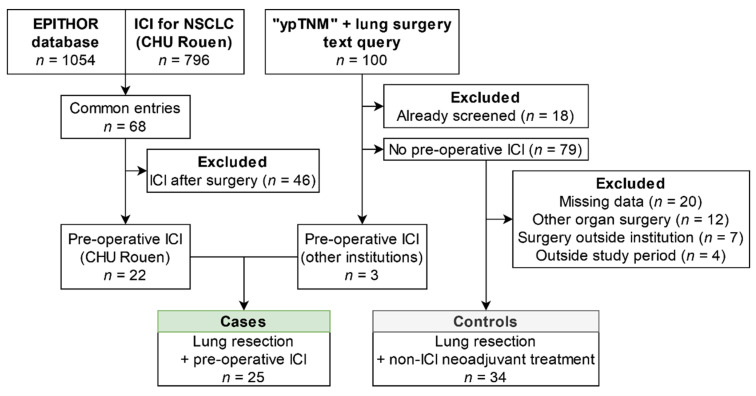
Flow chart of the study population. Abbreviations: ICI: immune checkpoint inhibitors; NSCLC: non-small-cell lung cancer; CHU Rouen: Rouen University Hospital.

A total of 25 patients fulfilled the inclusion/exclusion criteria for the ICI group, and 34 patients were included in the control group (Figure 1). 

The population characteristics of the participants are summarized in Table 1. The median age was 60.4 (56.0–67.0) years; 40 patients (67.8%) were male, with an underlying respiratory disease existing in 30 (50.9%) cases. Most patients were former or active smokers with an average tobacco exposure of 38.9 ± 16.0 pack-years. All patients underwent preoperative staging and pulmonary function tests (PFT), with an average FEV1 of 2.6 ± 0.7 L and a TLCO of 64.7 ± 15.0% of the predicted value. Exercise testing was performed in 27 (45.8%) patients, with a mean VO2 max of 17.7 (3.5) mL/kg/min. More patients underwent pulmonary rehabilitation prior to surgery in the ICI group (16 (64.0%) versus 12 (35.3%), *p* = 0.03). There was no difference in the cancer histology between the groups. All non-squamous NSCLC tested negative for EGFR mutation (assay: ABI Prism SNaPshot^®^, Life Technologies^®^, Carlsbad, CA, USA) as well as ALK and ROS1 translocation (immunohistochemistry using D5F3 and D4D6 clones, respectively).

At diagnosis, 13 (52.2%) of the ICI patients had oligometastatic disease versus 8 patients (23.5%) in the control group (*p* = 0.02). Local metastases treatments were performed in 19 (32.2%) patients in the study population: 11 (44.0%) in the ICI group and 8 (23.5%) in the control group, *p* = 0.16. These treatments were: surgical metastasis resection (*n* = 10, 16.9%), stereotactic radiosurgery (*n* = 11, 18.6%), total brain irradiation (*n* = 3, 5.1%), or conformational radiotherapy (*n* = 3, 5.1%). In the ICI group, 20 patients (80.0%) received pembrolizumab and 5 (20.0%) received nivolumab, with a median of five (2–18) infusions prior to surgical intervention. Surgery was performed after a median of 39 days (22–49) from the last infusion. ICIs were administered as first-line monotherapy in 14 (56.0%) patients, as part of a first-line combined chemo-immunotherapy regimen in 3 (12.0%) patients, and as a second- or third-line treatment following standard chemotherapy in 8 (32.0%) patients.

### 3.2. Tumour Characteristics and Surgical Interventions

Table 2 shows the comparison between the ICI and control groups regarding target lesion and surgical intervention characteristics. The tumour anatomical topography was not statistically different among the groups. Half of the tumours were proximal (52% and 50% in the ICI and control groups, respectively). The performed surgery was a lobectomy in 60% of the patients and a pneumonectomy in 25% of the patients. A minimally invasive approach was planned in 17/25 (80.0%) and 20/34 (58.8%) of the patients in the ICI and control groups, respectively. 

Indications for surgical intervention varied widely between groups (*p* < 0.001). In the control group, 20 (58.8%) patients were referred for surgery after a neoadjuvant treatment for resectable stage II-III disease, 5 (14.7%) underwent lung resection following the downstaging of initially unresectable stage III disease, 8 (23.5%) received surgery as part of the multimodal management of oligometastatic disease, and 1 (2.9%) had non-oligometastatic stage IV disease at diagnostic and underwent surgery for residual tumour ablation. In the ICI group, patients underwent surgery in the setting of a diffuse stage IV disease after prolonged partial response to ICIs (*n* = 8, 32.0%): three underwent surgery due to the suspicion of local disease oligoprogression, and five underwent surgery for residual tumor ablation. In seven patients (28.0%), surgery was performed after a neoadjuvant ICI treatment for resectable stage II-III disease; in six patients (24.0%), surgery was performed following the downstaging of previously unresectable disease; and four patients (16.0%) underwent surgery for the management of oligometastatic disease.

**Figure 2:**
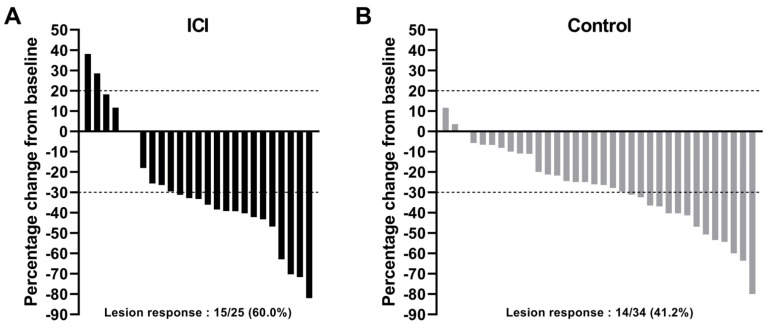
Waterfall plot of change in target lesion size between baseline and preoperative imaging in the ICI (**A**) or control (**B**) group. Lesion size refers to the largest diameter measured on CT-scan in the axial plane. ICI: received preoperative immune checkpoint inhibitors; control group: received standard pre-operative chemotherapy as part of an induction treatment.

When considering the size of the target lesion, pre-operative systemic treatment allowed for a similar reduction, with a median of 33.3% [9.0–42.8] and 26.3% [10.7–40.6] in the ICI and control groups, respectively (*p* = 0.56). The percentage change of the target lesion size between baseline and preoperative imaging is presented in Figure 2. 

### 3.3. Surgical, Perioperative, and Pathological Outcomes

Surgical, perioperative, and pathological outcomes are presented in Table 3. Operative times were similar in the ICI and control groups (180.6 ± 54.6 min and 192.8 ± 68.4 min, respectively, *p* = 0.81). Among the surgeries attempted with a minimally invasive approach, there were 6/17 (35.3%) and 4/20 (20.0%) conversions to thoracotomy in the ICI and control groups, respectively (*p* = 0.46). No intraoperative death occurred.

Operative report reviewing found surgeon-reported challenges in 17 cases (68.0%) and 21 cases (61.7%) in the ICI and control group, respectively (*p* = 0.83). The proportions of challenging dissections, tissular adhesions or bridles, and intraoperative vascular wounds were similar (Table 3). Tissue fibrosis or inflammation was reported in 10 (40.0%) patients in the ICI group. Of note, it was reported in 7/13 (53.8%) of proximal tumour resections and in 3/11 (27.3%) distal tumour resections (*p* = 0.24). In the control group, it was reported for one patient (2.9%) (*p* < 0.01). In three patients (12.0%) from the ICI group, tissue fibrosis led to a modification in the surgical procedure: i.e., the planned resection was not achieved in two patients (8.0%), or lymph node dissection was incomplete (*n* = 1, 4.0%). Of note, among these three patients, all of them had a pathological complete response and would remain in complete response at data cut-off.

There were no differences in the post-operative stay length or complications between the groups. There was no 90-day mortality. Post-operative complications were mostly grade I or II (42/52 complications (80.8%)). There were seven (28.0%) and nine patients (26.4%) who developed more than one complication in the ICI and control groups, respectively (*p* = 0.13). In total, 3 out of 25 (12.0%) and 6/34 (17.6%) patients had a grade ≥ III complication in the ICI and control groups, respectively. The severe complications (grade III and above) that were found in the ICI group were ruptured anastomosis (grade IIIb, *n* = 1), severe pneumonia (grade IIIa, *n* = 2), and hemothorax (grade IIIb, *n* = 1). In the control group, there were three cases of grade IIIa pneumonia, one hydropneumothorax (grade IIIa), one pneumothorax (grade IIIa), and one hemothorax (grade IVb).

**Figure 3:**
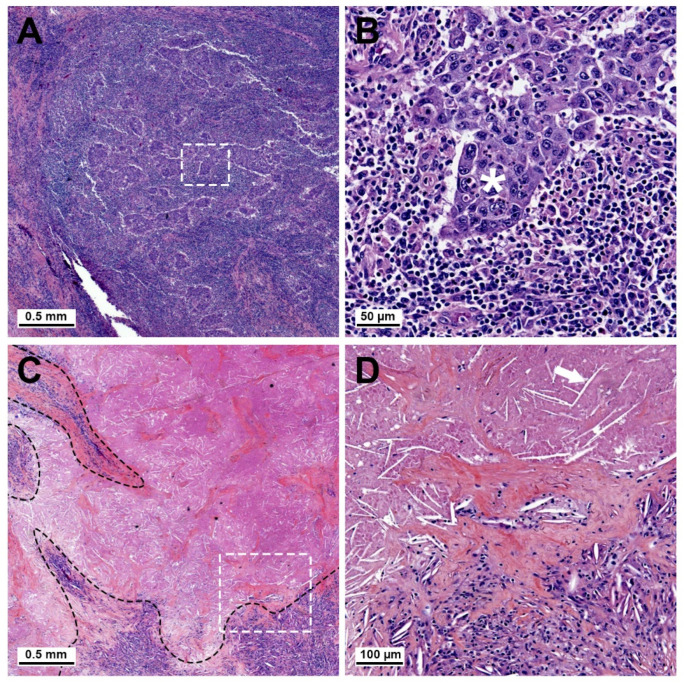
Representative slides for estimation of the residual tumor using hematoxylin and eosin staining in the ICI cohort. (**A**) Example of major pathological response (MPR) in one patient. Islets of residual viable tumor tissue are surrounded by a major inflammatory cellular infiltrate. White rectangle is magnified in (**B**) and shows a residual tumor islet with large atypical cells (white asterisk) surrounded by small mononucleated inflammatory cells. (**C**) Example of complete pathological response (pCR) in one patient, with zones of tissue fibrosis and inflammatory infiltrates (delineated in black) and large areas of tumor necrosis with no residual viable tumor. White rectangle is magnified in (**D**) and shows the demarcation between fibrotic, inflammatory tissue, and necrosis with cholesterol crystal imprints (arrow).

During the histopathology, a major pathological response (MPR), defined as a residual viable tumor (RVT) ≤ 10% on postoperative pathological examination, was observed in 12 patients (48.0%) in the ICI group and in 8 patients (23.5%) in the control group (*p* = 0.049), with pathological complete response (pCR) observed in 7 (28.0%) and 5 (14.7%), respectively (*p* = 0.21) (Figure 3). R0 resection was achieved in 96% and 91% of the patients, respectively. After surgery, a pathological downstaging was observed in 15 patients (60.0%) in the ICI group and 13 patients (38.2%) in the control group (*p* = 0.1).

### 3.4. Clinical Outcomes

**Figure 4:**
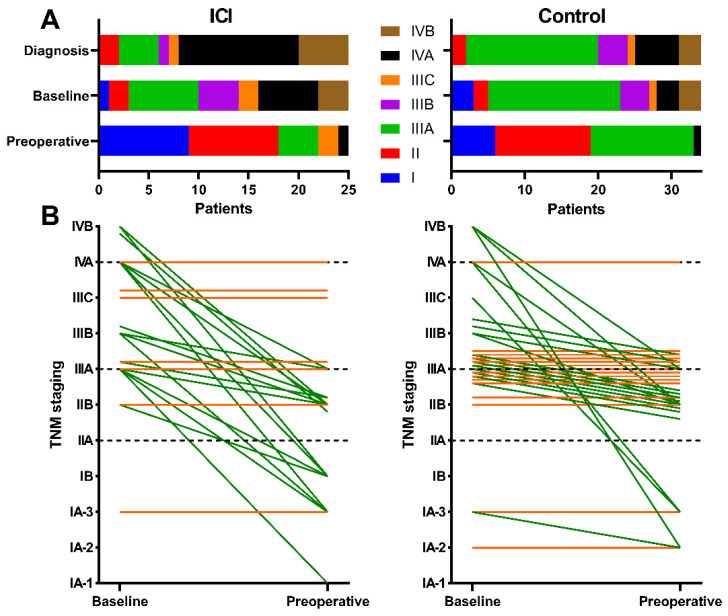
Evolution of clinical tumour staging in the study population. (**A**) Stacked bar chart displaying the spread of cTNM staging across patients from the ICI group at different stages of evaluation: at disease diagnosis, at ICI or chemotherapy baseline imaging, and on pre-operative evaluation. (**B**) Before-and-after graph of the evolution of cTNM cancer staging in each patient between baseline and preoperative evaluations in the ICI group (**left**) and the control group (**right**). Each line represents a single patient. Green lines signify downstaging. Orange lines represent patients without stage changes. Local treatments of oligometastases were taken into account where applicable.

Figure 4A shows the spread in clinical cancer staging (cTNM) in the ICI group at three timepoints: cancer diagnosis, baseline evaluation, and preoperative evaluation. Figure 4B displays the staging evolution of every patient between the last two timepoints; the cTNM stage decreased in 18 (75.0%) patients from the ICI group and in 18 (52.9%) patients from the control group.

**Figure 5:**
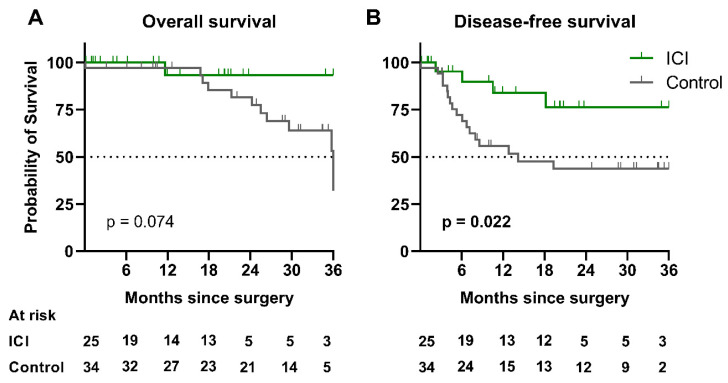
Three-year survival analyses in the study population. (**A**) Kaplan–Meier curves of 3-year overall survival in patients of the ICI group versus the control group. (**B**) Kaplan–Meier curves of 3-year disease-free survival in both groups. Abbreviation: ICI: immune checkpoint inhibitors.

**Figure 6:**
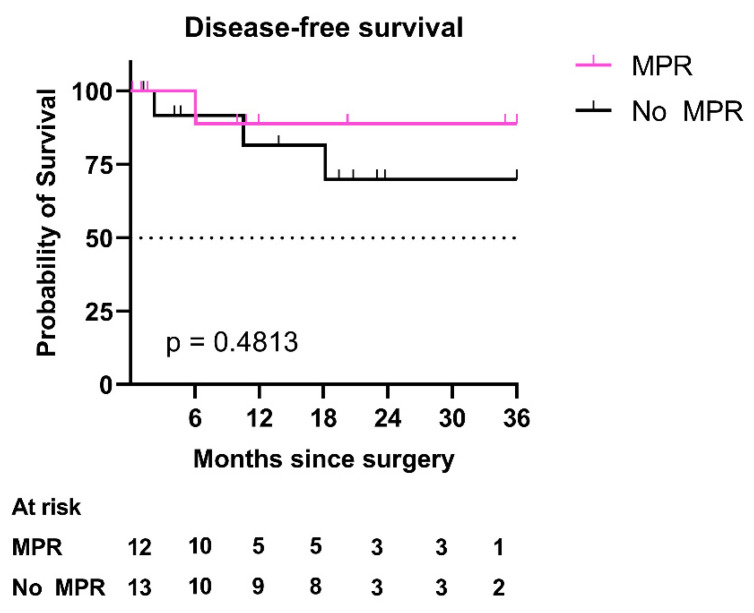
Three-year disease-free survival in the ICI group. Kaplan–Meier curves of 3-year disease-free survival in patients of the ICI group with MPR versus patients without MPR. Abbreviations: ICI: immune checkpoint inhibitors; MPR: major pathological response.

After a median follow-up of 21.0 [10.2–33.3] months, median disease-free survival (DFS) was not reached in the ICI group nor after a 14.3-month follow-up in the control group (*p* = 0.022), with a hazard ratio (HR) of 0.30 (CI 95% [0.13–0.71]). Median overall survival (OS) was not reached in the ICI group nor at 36.0 months in the control group (*p* = 0.074) (Figure 5). In the ICI group, 17 (68.0%) patients were alive showed no evidence of recurrence at the last follow-up. DFS in patients with MPR in the ICI group was not statistically different from those without MPR (HR = 0.45 (CI 95% [0.06–3.30], *p* = 0.4813) (Figure 6). 

**Figure 7:**
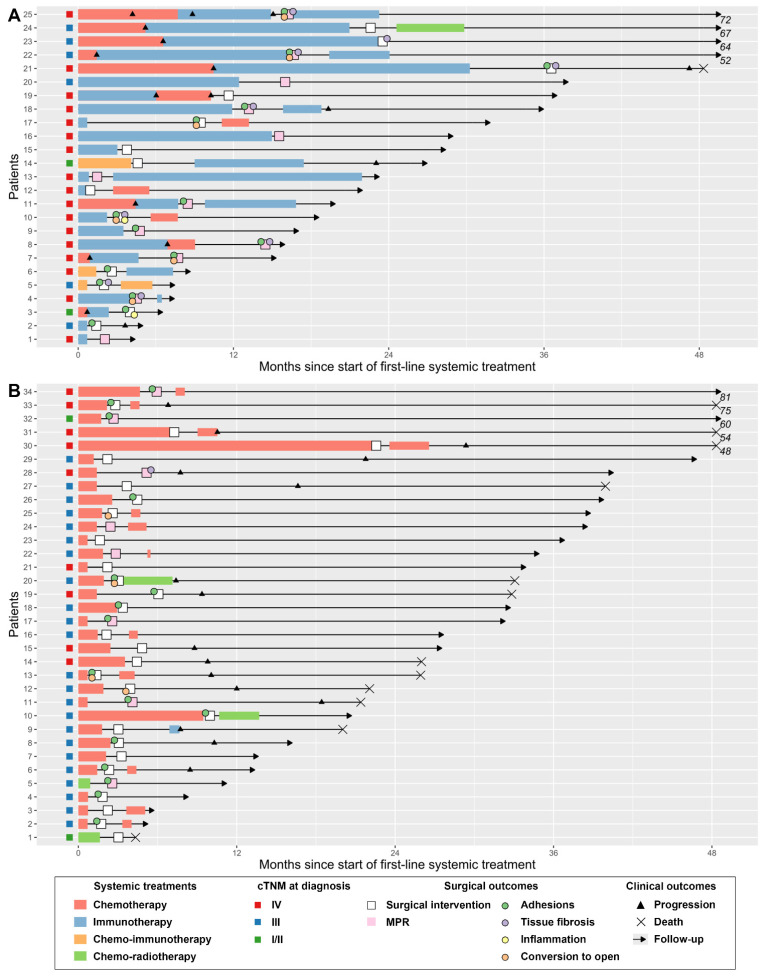
Swimmer plot showing duration of follow-up; systemic treatments administered; and surgical, clinical, and pathological outcomes. (**A**) Immune checkpoint inhibitors group (*n* = 25). (**B**) Control group (*n* = 34). Systemic treatments and clinical progressions were recorded exhaustively prior to surgery. Following surgery, first-line adjuvant treatment, date of first progression, and last follow-up were collected. Abbreviations: MPR: major pathological response.

The clinical courses and outcomes of patients in both groups are summarized in Figure 7. In the ICI group, following resection, 9 (36.0%) patients continued their ICI treatment, 3 (12.0%) received chemotherapy, and 1 (4.0%) received radiochemotherapy, while 12 (48.0%) did not receive adjuvant systemic treatment.

## 4. Discussion

In this study, we reported the surgical and clinical outcomes of 25 patients who underwent lung resection for NSCLC following treatment with ICIs. We compared their characteristics and outcomes to 34 patients who had received pre-operative chemotherapy without ICIs. In the ICI cohort, median disease-free survival was not reached after a median follow-up duration of 21 months. Surgery in these patients was often challenging because of adhesions, inflammation, and tissue fibrosis. These events were more frequently reported in the ICI group, especially in tumours having a close contact with the hilum. Nevertheless, perioperative complications were similar in both cohorts, and the 90 days mortality was 0% in both groups.

To the best of our knowledge, this is the first case–cohort study evaluating the safety and outcomes of lung resections in NSCLC patients after treatment with ICI. One strength of our study was its real-world setting, as we chose to include all consecutive patients who had received pre-operative ICI, regardless of indication. 

The patients in our study were younger than those usually reported for NSCLC patients in France. This is probably related to the design of our study, by which only patients who had lung resection and preoperative medical treatment were eligible. 

The morbidity and mortality rates in patients undergoing lung resection following conventional chemotherapy or radiochemotherapy as a neoadjuvant treatment are well studied and have decreased over recent years, owing to advances in patient selection, surgical techniques, and the optimization of neoadjuvant drug regimens [9,23]. Only twelve years ago, pneumonectomy after induction chemotherapy for NSCLC was still deemed controversial because of high mortality rates and sometimes unclear survival benefits [24], with series reporting mortality rates of up to 26% [25]. However, these results rapidly improved; in 2005, Van Schil et al. reported the outcomes of 167 patients included in the surgical arm of the EORTC 08941 trial investigating chemotherapy plus surgery versus radiochemotherapy in IIIA patients, with a 90-day mortality of 8.7% [26]. In this study, 81% of patients experienced a post-operative complication, mainly in the pulmonary and pleural spaces (27.9% and 18.4%, respectively). Six years later, Barnett et al. reported a series of 549 patients undergoing surgery after induction therapy, all of whom had received chemotherapy (17% had also received radiation therapy) [23]. Complications occurred in 46% patients, with grade 3 complications or higher in 23%. In hospital mortality reached 1.8%, with one death following a pneumonectomy (1/30). Multivariate analysis showed that perioperative morbidity was associated with poor lung function. In their 2017 review of the Society of Thoracic Surgeons General Thoracic Surgery Database, Boffa et al. found a postoperative mortality of 2.5% with induction compared to 1.9% with upfront surgery in 1535 patients with stage IIIA NSCLC [27]. Overall, there is a dramatic improvement in mortality rates after lung resection following induction therapy. 

With regard to preoperative ICI treatment, in 2018, Bott et al. reported results from 19 patients with NSCLC or melanoma who had undergone lung resection after treatment with an ICI [15]. In this study, most interventions were wedge resections (50%). Fifteen patients (68%) had residual viable tumour (RVT) upon final pathological assessment. There was no perioperative mortality and one grade > 3b complication. The potential effects of ICI on the technical aspects of lung resection were investigated, and the authors noted that out of eight lobectomies, dense tissue adhesion was reported in four. In one case, the right upper lobe bronchus and truncus branch of the pulmonary artery were fused, and dissection was reported to be extremely challenging. The same team also reported a series of 20 patients who had undergone the resection of localized NSCLC after pre-surgery treatment with nivolumab [18]. Among the 13 procedures attempted via a minimally invasive approach, 7 (54%) required thoracotomy, owing mainly to fibrotic adhesions. The median operating time was 228 min. A total of nine patients (45%) had MPR, and eight (40%) had pathologic downstaging. Finally, Romero Román et al. reported 41 patients who had undergone lung resection for stage IIIA NSCLC following 3 cycles of neoadjuvant treatment with paclitaxel, carboplatin, and nivolumab [16]. The conversion rate was 19% (*n* = 4). R0 resection was achieved in all patients, and downstaging was observed in 90.2%, with MPR in 34 patients (82.9%). The complications were mainly low-grade and mostly consisted of persistent air leak (*n* = 8), pneumonia (*n* = 5), and arrythmia (*n* = 4).

In our study, tissue fibrosis and inflammation were reported in 40.0% of patients after ICI treatment versus 2.9% after chemotherapy. As a result, planned minimally invasive surgery was converted to thoracotomy in 6/17 patients. This mainly occurred for tumour resection involving the peri-hilar area. Nevertheless, morbidity was in line with data from trials evaluating standard induction chemotherapy, and no patient died within 90 days of surgery. Eighteen patients (72.0%) had RVT upon final pathological examination in the ICI group, with 44% exhibiting MPR, while 60% had pathologic downstaging. 

Most of the patients from the ICI group had stage IV disease at diagnosis (17/25, 68%). This illustrates a paradigm shift in the role of surgery in NSCLC management. In fact, the efficacy of ICI treatment allows for the long-term control of stage IV NSCLC in some patients, which drives clinicians to go beyond the standard indications of surgery. However, the therapeutic value of resecting residual primary or metastatic tumours remains controversial. In practice, the term “resectable” not only refers to technical considerations but also to cases when resection can be expected to favorably impact prognosis [28], and in our experience, residual tumour resection may help to achieve long-term disease control in the context of ICI treatment. In this study, we showed encouraging results in terms of overall postoperative and disease-free survival in ICI patients despite their “unresectable” status at diagnosis. However, the extrapolation of this finding is limited by the heterogeneity of the ICI group regarding clinical situations. 

Recent findings from large-scale clinical trials investigating neoadjuvant PD-1 blockade for the treatment of resectable NSCLC have shown encouraging results [8,9]. Therefore, ICIs are bound to become part of the multi-modal management of resectable NSCLC. The small sample size of our study did not allow us to perform pair-matched statistical analysis. Additional data regarding surgical outcomes in these patients is necessary, and phase 3 trials with combined immuno-chemotherapy induction will probably yield valuable information related to this clinical situation [29] (NCT02998528—CheckMate 816, NCT03456063—IMpower30, NCT03425643—KEYNOTE-671) [30,31,32].

## 5. Conclusions

In our small, retrospective study, ICI-based treatment before surgery did not impact lung resection safety compared to chemotherapy alone in advanced or metastatic NSCLC patients. This conclusion should be limited to patients with clinical characteristics that are comparable to those of the patients included in our trial.

This study supports the feasibility and safety of lung resection in patients following preoperative treatment with ICIs. We showed that tumour tissue dissection involving the perihilar area is more challenging in this context; however, this did not translate into higher mortality or morbidity.

## Figures and Tables

**Table 1:** Preoperative patient characteristics.

Variable	Total Population (*n* = 59)	ICI Group (*n* = 25)	Control Group (*n* = 34)	*p*
Age, years	60.4 (56–67)	60.0 (54.6–62.8)	62.7 (57.6–67.7)	0.26
BMI, kg/m^2^	24.7 ± 4.6	25.2 ± 4.7	24.3 ± 4.6	0.27
**Gender**				0.98
Male, *n* (%)	40 (67.8)	17 (68.0)	23 (67.6)	
Female, *n* (%)	19 (32.2)	8 (32.0)	11 (32.4)	
**Tobacco status**				
Active or former smoker, *n* (%)	55 (93.2)	23 (92.0)	32 (94.2)	>0.99
Exposure, pack-years	38.9 ± 16.0	37.8 ± 21.1	39.6 ± 11.3	0.51
**Comorbidities**				
ASA score	2 (2–3)	2 (2–3)	2 (2–3)	0.70
COPD, *n* (%)	25 (42.4)	11 (44.0)	14 (41.2)	0.82
Asbestos exposure, *n* (%)	9 (15.3)	3 (12.0)	6 (17.6)	0.72
Other respiratory disease *, *n* (%)	5 (8.5)	3 (12.0)	2 (5.9)	0.64
Arterial hypertension, *n* (%)	22 (37.3)	10 (40.0)	12 (35.3)	0.79
Diabetes, *n* (%)	5 (8.5)	3 (12.0)	2 (5.9)	0.64
Dyslipidemia, *n* (%)	12 (20.3)	8 (32.0)	4 (11.8)	0.10
History of thoracic surgery, *n* (%)	3 (5.1)	1 (4.0)	2 (5.9)	>0.99
**Pulmonary function testing**, *n* (%)	59 (100)	25 (100)	34 (100)	-
FEV1, L	2.6 ± 0.7	2.5 ± 0.6	2.6 ± 0.8	0.73
FEV1, % predicted	86.4 ± 16.7	85.5 ± 14.8	87.2 ± 18.2	0.76
FVC, L	3.6 ± 0.9	3.6 ± 0.9	3.6 ± 0.9	0.78
FVC, % predicted	99.7 ± 14.5	100.0 ± 14.3	99.5 ± 14.8	0.94
TLC, L	6.43 ± 1.3	6.3 ± 1.4	6.6 ± 1.3	0.39
TLC, % predicted	105.2 ± 14.9	105.2 ± 14.2	105.0 ± 15.7	0.89
FEV1/FVC, %	69.2 ± 9.7	69.4 ± 8.7	69.1 ± 10.4	0.85
TLCO, % predicted	64.7 ± 15.0	64.9 ± 15.8	64.6 ± 14.6	0.79
**Exercise testing**, *n* (%)	27 (45.8)	10 (40.0)	17 (50.0)	0.60
VO2 max, mL/kg/min	17.7 ± 3.5	17.6 ± 2.1	17.8 ± 4.2	0.78
Pulmonary rehabilitation, *n* (%)	21 (35.6)	16 (64.0)	12 (35.3)	0.03
**Histopathology**				
Adenocarcinoma, *n* (%)	37 (62.7)	18 (72.0)	19 (55.9)	0.39
Squamous cell carcinoma, *n* (%)	20 (33.9)	6 (24.0)	14 (41.2)	
Carcinoma NOS, *n* (%)	2 (33.9)	1 (4.0)	1 (2.9)	
PD-L1 expression available, *n* (%)	40 (67.7)	20 (80.0)	20 (58.8)	0.08
0%	13 (22.0)	3 (12.0)	10 (29.4)	<0.01
1–49%	6 (10.2)	1 (4.0)	5 (14.7)	
≥50%	18 (30.5)	16 (64.0)	5 (14.7)	

Abbreviations: BMI: body mass index; COPD: chronic obstructive pulmonary disease; ASA: American Society of Anesthesiologists; FEV1: forced expiratory volume in one second; FVC: forced vital capacity; TLC: total lung capacity; TLCO: diffusion capacity of carbon monoxide; VO2 max: maximal oxygen uptake. * Other respiratory diseases: obstructive sleep apnea in 3 patients (5.1%), asthma in 1 (1.7%), nontuberculous mycobacteria in 1 (1.7%). As per Bonferroni correction for 27 comparisons, a *p* < 0.0019 indicates statistical significance here.

**Table 2:** Characteristics of surgical procedures.

Variable	ICI Group (*n* = 25)	Control Group (*n* = 34)	*p*
**Location of target lesion**			0.08
RUL, *n* (%)	7 (28.0)	15 (44.1)	
LUL, *n* (%)	11 (44.0)	6 (17.6)	
RLL, *n* (%)	2 (8.0)	8 (23.5)	
LLL, *n* (%)	5 (20.0)	5 (14.7)	
**Topography**			0.38
Proximal (juxta-mediastinal), *n* (%)	13 (52.0)	17 (50.0)	
Distal, *n* (%)	11 (44.0)	12 (35.2)	
Sulcus tumour, *n* (%)	1 (4.0)	5 (14.7)	
Size of target lesion on imaging *			
Baseline evaluation, mm	58.6 ± 26.0	63.0 ± 25.9	
Preoperative evaluation, mm	40.0 ± 21.8	44.3 ± 20.9	
Evolution, %	−33.3 (9.0–42.8)	−26.3 (10.7–40.6)	0.56
**Type of resection**			0.41
Single lobectomy, *n* (%)	15 (60.0)	21 (61.8)	
Pneumonectomy, *n* (%)	6 (24.0)	10 (29.4)	
Bilobectomy, *n* (%)	2 (8.0)	3 (8.8)	
Segmentectomy, *n* (%)	2 (8.0)	0	
**Surgical approach**			0.58
VATS only, *n* (%)	11 (42.0)	16 (47.1)	
Thoracotomy, *n* (%)	14 (58.0)	18 (52.9)	
Robot-assisted surgery, *n* (%)	7 (28.0)	5 (14.7)	
**Surgery setting**			<0.005
After neoadjuvant treatment, *n* (%)	7 (28.0)	20 (58.8)	
Downstaging, *n* (%)	6 (24.0)	5 (14.7)	
Residual tumoral ablation, *n* (%)	8 (32.0)	1 (2.9)	
Oligometastatic disease management, *n* (%)	4 (16.0)	8 (23.5)	

Abbreviations: RUL: right upper lobe; LUL: left upper lobe; RLL: right lower lobe; LLL: left lower lobe; VATS: video-assisted thoracoscopy. * Size of lesion refers to the largest diameter measured on CT-scan in the axial reconstruction plane. As per Bonferroni correction for 27 comparisons, a *p* < 0.008 indicates statistical significance here.

**Table 3:** Comparison of surgical, perioperative, and pathological outcomes.

Variable	Preoperative ICI (*n* = 25)	Control (*n* = 34)	*p*
**Surgical outcomes**			
Operative time, min	180.6 ± 54.6	192.8 ± 68.4	0.81
Conversion after VATS, *n* (%)	6/17 (35.3)	4/20 (20.0)	0.46
Intraoperative mortality, *n* (%)	0	0	-
**Surgeon-reported difficulties**			
Challenging dissection, *n* (%)	7 (28.0)	10 (29.4)	0.91
Adhesions, *n* (%)	15 (60.0)	16 (47.1)	0.73
Tissue fibrosis or inflammation, *n* (%)	10 (40.0)	1 (2.9)	<0.0004
Intraoperative vascular wound, *n* (%)	3 (12.0)	4 (11.8)	>0.99
Procedure adapted during surgery *, *n* (%)	3 (12.0)	2 (5.9)	0.64
**Perioperative outcomes**			
Total hospital LOS, days	7 (5–11)	7 (6–9)	0.74
ICU LOS, days	1 (0–1]	1 (0–2)	0.79
90-day mortality, *n* (%)	0	0	-
**Perioperative complications**—any grade	12 (48.0)	16 (47.1)	0.94
Persistent air leak, *n* (%)	4	6	-
Pneumonia, *n* (%)	6	4	-
Recurrent nerve palsy, *n* (%)	2	5	-
Respiratory failure, *n* (%)	2	3	-
Persistent pleural effusion, *n* (%)	2	0	-
Intrathoracic bleeding, *n* (%)	1	3	-
Arrythmia, *n* (%)	2	1	-
Atelectasis, *n* (%)	0	3	-
Sepsis, *n* (%)	1	1	-
Anastomosis failure, *n* (%)	1	0	-
Acute urinary retention, *n* (%)	0	1	-
**Pathological outcomes**			
R0 achieved, *n* (%)	24 (96.0)	31 (91.2)	0.63
RVT present, *n* (%)	18 (72.0)	29 (85.2)	0.33
Major pathological response °, *n* (%)	12 (44.0)	8 (23.5)	0.05
pCR, *n* (%)	7 (28.0)	5 (14.7)	0.21

Abbreviations: LOS: length of stay; ICU: intensive care unit; RVT: residual viable tumour; pCR: pathological complete response. * Procedure adapted during surgery: difficulties encountered during intervention caused a modification in surgery scope compared to preoperative plans. ° Major pathological response was defined as a RVT ≤ 10%. As per Bonferroni correction for 14 comparisons, a *p* < 0.0036 indicates statistical significance here.

## Data Availability

The data presented in this study are available on request from the corresponding author. The data is not publicly accessible in a repository due to privacy considerations.
